# Significant room for improvement in the prehospital assessment and treatment of acute abdominal pain: a retrospective observational study

**DOI:** 10.1186/s13049-025-01328-z

**Published:** 2025-01-27

**Authors:** Rasmus Bjerén, Carl Magnusson, Johan Herlitz, Denise Bäckström

**Affiliations:** 1 Center for Research and Development, Region Gävleborg, Gävle, Sweden; 2 Department of Prehospital Emergency Care, Sahlgrenska University Hospital, Gothenburg, Sweden; 3 Center for Prehospital Research, Faculty of Caring Science, Work Life and Social Welfare, University of Borås, Borås, Sweden; 4 Department of Biomedical and Clinical Sciences, Linköping University, Linköping, Sweden

**Keywords:** Emergency medical services, Abdominal pain, Pain management, Pain measurement, Acute pain, Pain

## Abstract

**Background:**

Acute abdominal pain (AAP) is a common reason for calling emergency medical services (EMS). Despite the widely acknowledged importance of effective prehospital pain management, described by patients as crucial regardless of any other factor, studies on prehospital pain management in AAP patients are limited and suggest room for improvement. This is particularly relevant given the long-standing controversy surrounding the use of analgesia in AAP patients before a final diagnosis is made, which may still influence the prehospital pain management.

**Methods:**

A retrospective cohort study of pain management in EMS patients with AAP in a central Swedish region. The region had a population density of 15.7 inhabitants per square kilometer spread over a mix of small urban and rural settings. Patient records were manually reviewed and scanned for written assessments or numeric ratings of pain. The analysis focused on proportions of assessment, treatment and reassessment of pain as well as median pain intensity, pain reduction and proportion of patients with a low last recording of pain.

**Results:**

816 patients were included. Pain was assessed in 55% (*n* = 447) of all cases. The median initial pain intensity was eight units (IQR 6.0–9.0) on the Numerical Rating Scale (NRS), and 90% (*n* = 403) of the assessed patients experienced moderate or severe pain. Of those, 62% (*n* = 249) received pharmacological treatment. In 50% (*n* = 158) of all cases receiving treatment, pain was reassessed afterwards. The median pain reduction was four units (IQR 2.0–5.0) on the NRS scale. Among all cases, 10% (*n* = 84) had a last recorded pain assessment indicating low pain.

**Conclusions:**

Significant room for improvement in the prehospital management of acute abdominal pain was found. The proportions of pain assessment, treatment and reassessment were low with nine out of ten patients leaving prehospital care with unknown, moderate or severe pain. Among the cases where pain assessment, treatment and reassessment were made and recorded, four out of five patients experienced significant pain relief, indicating the potential of better prehospital pain management.

## Background

Acute abdominal pain (AAP) is one of the most common reasons for calling Emergency Medical Services (EMS), accounting for 11% of all assignments [1]. It is the most common reason for visiting the emergency department (ED) [2]. Pain is defined by the International Association for the Study of Pain as “an unpleasant sensory and emotional experience associated with actual or potential tissue damage, or described in terms of such damage” [3] and had a prevalence of 42% among EMS patients in a previous study [4]. Moderate to severe pain has been reported in 26–28% of all patients [4, 5]. Among patients diagnosed within ICD-10 chapter XI (“diseases of the digestive system”) and XIV (“Diseases of the genitourinary system”), many of whom are likely to present with abdominal pain, the proportions of moderate to severe pain are notably higher, at 41% and 34% respectively [5].

Effective pain management is a key factor for enabling high-quality person-centered care. It is crucial for the patient regardless of almost any other factor [6] and has been increasingly recognized as a human right in recent years [7, 8]. Pain management has been identified by the European Society for Emergency Medicine (EUSEM) as one of the most important contributions of emergency care [9] and has also been defined as a key quality outcome measure in EMS organizations [10]. To enable successful pain management, a good clinician-patient relationship that handles the patient’s needs, expectations and beliefs is crucial. Patients describe pain management as highly meaningful, from establishing trust in the caring relationship to practical aspects of prehospital care such as facilitating movement to the ambulance [6]. Other positive effects described include a reduced risk of complications such as chronic pain [11] as well as less adverse psychological effects such as anxiety or inability to sleep [12].

Pain management can be divided into two cornerstones: pain assessment and pain treatment [9, 13]. Assessment can be performed via a plethora of scales, with different strengths and limitations. The Numerical Rating Scale (NRS) has been described as a practical tool for a time-pressured environment such as prehospital care, since it offers a more sensitive measurement than verbal descriptions of pain while still being easy to use [9, 14]. Treatment alternatives include pharmacological and nonpharmacological options. The latter ones can for example be positioning, relaxation, or the application of warmth or cold as well as caring measures such as timely information and reassurance [6, 15]. Studies indicate that a pain reduction of between one and two NRS units is required to be perceived as clinically significant in the ED [16, 17].

Despite the importance being widely acknowledged, several studies have illuminated room for improvement in pain management within the EMS. Those studies indicate that less than 40% of all EMS patients in pain receive pain treatment, with low proportions of pain assessment and reassessment [18, 19]. Studies of prehospital pain management in patients with AAP are rare, while EMS treatment may still be effected by the old controversy surrounding the use of analgesia to those patients before the final diagnosis has been determined [20]. To the best of our knowledge, there are currently no studies examining prehospital pain management in patients with AAP in sparsely populated areas. This study aims to explore prehospital pain management and its effect on AAP patients in a such region.

## Methods

### Design

The study is a retrospective cohort study. Patient records for adult EMS patients classified by the ambulance clinician (AC) as abdominal pain were manually reviewed. The STROBE checklist was used throughout the research process to maintain publication quality [21].

### Setting

The study was performed in a central Swedish region with a catchment area of 18.191 km^2^ and a population of 287.334 people by the end of 2021 [22], i.e. a population density of 15.8 inhabitants per square kilometer. The population was spread among cities and sparsely populated areas divided into 10 municipalities, five of which were populated by more than 20.000 people [23]. In 2021, a total of 42.479 assignments were executed by 22 ambulances and one single responder, geographically spread over six main stations and six auxiliary locations. Ambulances were publicly funded and staffed with two ACs of which at least one was a registered nurse [24]. Twenty-one percent of the nurses had a one-year postgraduate program specializing in prehospital care [25]. All primary EMS assignments included the establishment of a priority using the South African Triage Scale (SATS). The triage consisted of three parts: a score calculated with the Triage Early Warning Score, a set of discriminators resulting in a higher priority, including one of particular interest to this study indicating pain with NRS ≥ 7, and a part where a higher priority can be set on the basis of AC judgement [26]. The part with highest priority out of the three became the patient’s overall triage priority, described as one out of four colors; green for lowest acuity (routine), yellow (urgent), orange (very urgent) or red for highest acuity (emergency) [27]. The electronic patient record (EPR) also included a nonmandatory field for NRS allowing multiple registrations, as well as several free text fields where notes such as a verbal pain assessment could be documented in free text. The regional EMS guidelines [28] advised thorough pain anamnesis and repeated pain assessments and documentation of pain using the NRS or Visual Analogue Scale (VAS). Pharmacologic treatment of pain was suggested from NRS ≥ 4, with paracetamol per oral (p.o.) or intravenous (i.v.) as the base treatment if time allowed, supplemented by esketamine i.v./intranasal (i.n.)/intramuscular (i.m.) as well as morphine i.v. or sufentanil i.n. Nurses with a degree of anesthesiology could also use sufentanil i.v. and alfentanil i.v. For biliary colic or renal colic pain, diclofenac i.m. was specifically advised. Furthermore, the guidelines allowed for and sometimes recommended combinations of the mentioned drugs during the same caring encounter, for example esketamine followed by morphine and paracetamol. Nonpharmacological guidelines were limited to finding a comfortable position for the patient during transport [29]. In addition to the written guidelines, a primary care physician was available by phone on a 24/7 basis for individualized prescriptions or advice.

### Sampling

**Fig. 1:**
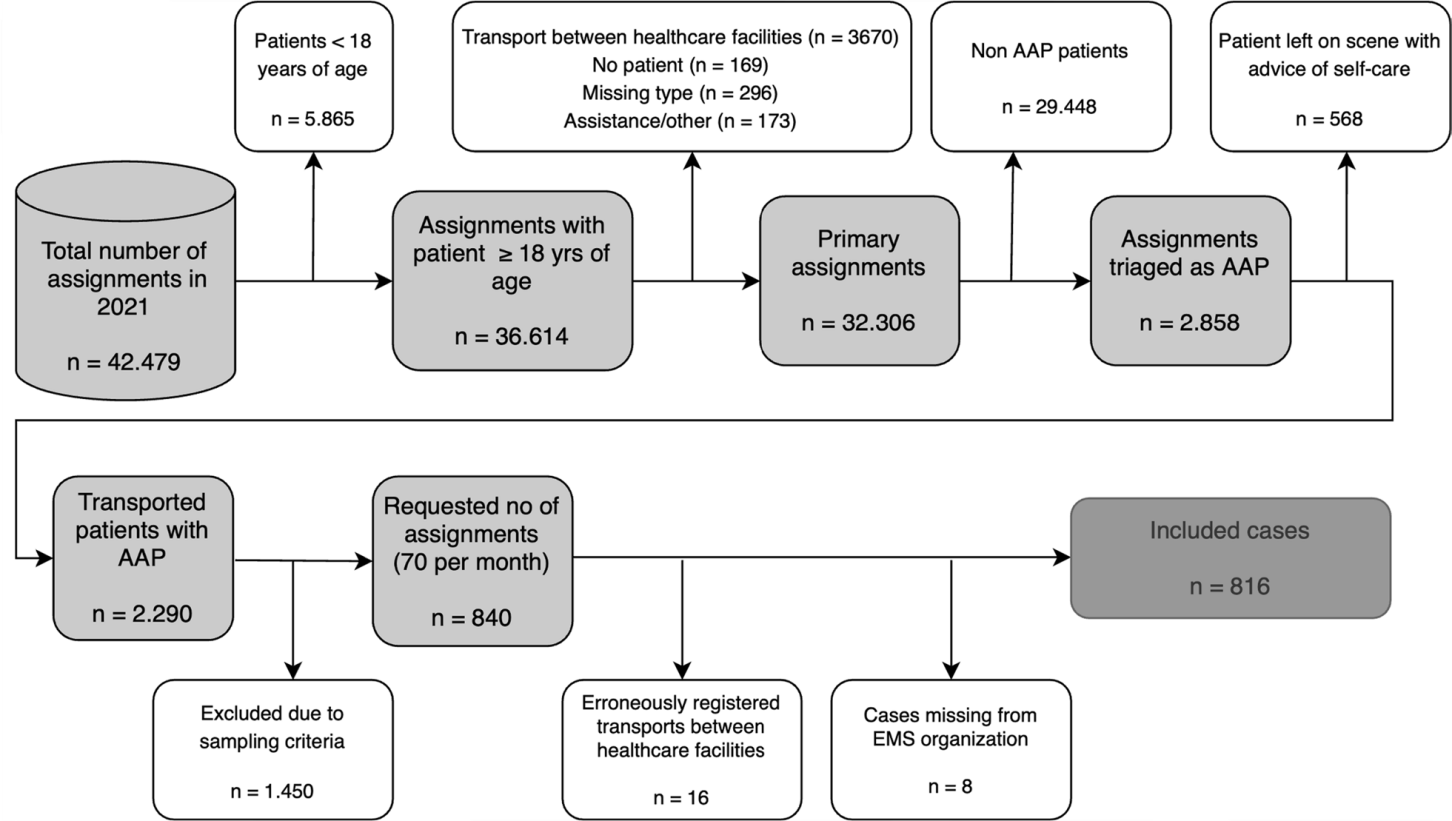
Sampling and excluded/missing cases

Data was collected using consecutive convenient sampling. A target of 840 patient records was set and the first 70 records of each month in 2021 fulfilling the inclusion criteria were selected, to achieve an even distribution throughout the year. The inclusion criterion was patients on primary assignments classified by the AC as abdominal pain. The exclusion criteria were*:* (1) Patients < 18 years of age, (2) transport between healthcare facilities, (3) assistance to another ambulance, and (4) assignments where the patient was left on scene with advice of self-care. See Fig. 1.

### Data collection

The EMS organization provided data based on the inclusion and exclusion criteria and the EPRs were read in full. Characteristics such as priority, times, vital signs and triage along with the following pain-related variables were recorded: (1) pain assessed (yes/no), (2) pain intensity before treatment (first recording), (3) pharmacological pain treatment (yes/no), (4) other pain treatment (yes/no) (5) type of pharmacological pain treatment (medication name and administration route), (6) type of other pain treatment, (7) pain reassessed after treatment (yes/no) and (8) pain intensity after treatment (last recording available). The synthesis of the patient’s pain level was derived primarily from an NRS value. In the absence of this, verbal pain assessments documented in free text were used. Where neither was available, usage of the triage discriminator indicating NRS ≥ 7 was interpreted as severe pain. The synthesis was established based on the following interpretations: Low pain = NRS 0–3 or free text descriptions of a patient with clearly bearable pain or who was mostly unaffected by the pain. Moderate pain = NRS 4–6 or free text descriptions of a patient clearly affected by pain, while still describing it as manageable and with descriptions of a reasonably calm behavior. Severe pain = NRS 7–10, use of triage discriminator” Pain NRS ≥ 7″ or free text descriptions such as “unbearable pain”, “screaming”, “climbing the walls” or other means of describing a patient with intense pain. Some reassessments were recorded as free text evaluations of treatment that did not specify a resulting level of pain, such as “morphine i.v. with good effect”. In those situations, pain was considered reassessed, but no resulting pain intensity or synthesis were recorded. Pharmacological treatment recordings were retrieved from a dedicated listing in the EPR, whereas nonpharmacological treatment was parsed from free text.

### Analysis

The analysis focused on pain assessment, treatment, and reassessment. Proportions of assessment, NRS usage and median pain intensity, as well as proportions and types of pain treatment, were calculated. The treatment effect was evaluated by calculating the median reduction in the NRS score and pain synthesis as well as the proportion of patients who experienced a significant pain reduction, defined as two or more NRS units. Furthermore, we assumed that satisfactory pain management, as perceived by the patient, is closely associated with reaching a low pain intensity during the prehospital care episode, irrespective of whether this occurs through iterative treatment and reassessment or if pain is determined as low from the beginning. Accordingly, we calculated the proportion of patients with a low last recording of pain, both in the group of cases with at least one pain assessment and in the group of all cases. For vital signs, cutoff values for normal intervals were fetched from the Swedish national guideline Vårdhandboken [30], the Triage Early Warning Score [26] and local guidelines. For statistical analysis, SPSS Statistics version 28 (IBM Corp., Armonk, NY, USA) was used.

### Ethical considerations

This study was performed in accordance with Swedish guidelines on good research practices [31] and the principles outlined in the World Medical Association’s (WMA) Declaration of Helsinki [32]. It was approved by the Swedish Ethical Review Authority, decision no 2022–00926-01. Ensuring confidentiality and protection of the patients’ personal data was considered the most important ethical consideration. However, owing to the retrospective nature of the study, informed consent was waived, and the data was instead pseudoanonymized from the EMS organization.

## Results

Among the 840 selected records, a total of 816 were included in the final analysis. See Fig. 1.

### Characteristics

The patient sex was female in 56% (*n* = 457) of the cases. The median age was 64 years (IQR 41–79). In 24% (*n* = 195) of all cases the highest priority, priority 1, was assigned at the dispatch center. Priorities 2 and 3 represented 72% (*n* = 585) and 4% (*n* = 36) respectively. The median delay from dispatch until arrival of the ambulance was 13 min (IQR 9–20 min), the median time on scene was 18 min (IQR 12–25 min) and the median transport time was 19 min (IQR 9–37 min). The SATS triage priorities and the proportions of patients with altered vital signs are presented in Table 1. While 11% (*n* = 89) were transported to primary care and 88% (*n* = 719) to the ED, 1% (*n* = 8) of the patients had an unclear transport destination.

**Table 1:** Triage and vital signs (n = 816)

SATS triage	*n*	Percent %	*n* missing
Green (routine)	314	39.4	20
Yellow (urgent)	236	29.6	
Orange (very urgent)	232	29.1	
Red (emergency)	14	1.8	

Altered vital signs	*n*	Percent %	*n* missing
RR < 12 / min	2	0.2	12
RR > 20 / min	176	21.9	
SpO_2_ < 94%	89	11.5	43
Pulse < 51 / min	9	1.1	3
Pulse > 100 / min	148	18.2	
Sys BP < 101 mmHg	35	4.3	11
Sys BP > 199 mmHg	14	1.7	
Altered LOC (aVPU)	20	2.5	11
Temp ≤ 36 °C	56	6.9	6
Temp > 38 °C	58	7.2	

SATS, South African triage scale; RR, respiratory rate; SpO_2_, peripheral oxygen saturation; Sys BP, systolic blood pressure; LOC, level of consciousness; aVPU, alert, verbal, pain, unresponsive; Temp, body temperature in degrees celsius

**Table 2:** Initial pain assessment, intensity and synthesis (n = 816)

Initial pain assessment	*n*	Percent %	*n* missing
Pain assessed	447	54.8	0

Pain scale used	*n*	Percent %	*n* missing
NRS	339	41.5	3
Free text	66	8.1	
SATS discriminator	39	4.8	

Initial pain intensity^a^	Median	IQR	*n* missing
NRS	8.0	6.0–9.0	0

Initial pain synthesis^b^	*n*	Percent %	*n* missing
Low pain	43	9.6	0
Moderate pain	89	20.0	
Severe pain	314	70.4	

NRS, Numeric Rating Scale; SATS, South African triage scale

^a^Among cases with NRS assessment (*n* = 339)

^b^Among cases with pain assessment (*n* = 447)

### Pain assessment and pain intensity

Pain was assessed in 55% (*n* = 447) of all cases. The NRS scale usage rate was 42% (*n* = 339). Among the cases where NRS were used the median pain intensity at first rating was eight units (IQR 6.0–9.0). Severe pain was found in 70% (*n* = 314) of the cases (Table 2).

### Pain treatment

**Fig. 2:**
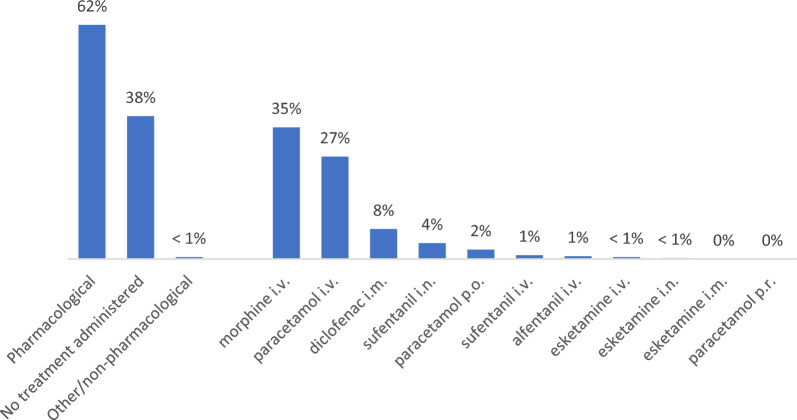
Pain treatment and treatment alternatives used among patients with moderate or severe initial pain (n = 403)

Pharmacological treatment of pain was given in 39% (*n* = 315) of all cases, whereas other types of treatment were very rarely used (*n* = 2). The two most frequently used treatment options were morphine i.v. (20%) and paracetamol i.v. (17%) (Table 3). In cases where the patient had severe or moderate pain, pain treatment was more common, as illustrated in Fig. 2.

**Table 3:** Pain treatment among all cases (n = 816)

Pain treatment	*n*	Percent %
Pharmacological	315	38.6
Other	2	0.2

Treatment options and routes	*n*	Percent %
Morphine i.v	162	19.9
Esketamine i.v	3	0.4
Esketamine i.n	1	0.1
Esketamine i.m	0	0.0
Sufentanil i.n	18	2.2
Sufentanil i.v	4	0.5
Diclofenac i.m	42	5.1
Alfentanil i.v	3	0.4
Paracetamol p.o	17	2.1
Paracetamol i.v	142	17.4
Paracetamol p.r	0	0.0

i.v., intravenous; i.m., intramuscular; i.o., intraosseous; p.o., per oral; i.n., intranasal; p.r., per rectum

### Reassessment of pain after treatment

**Fig. 3:**
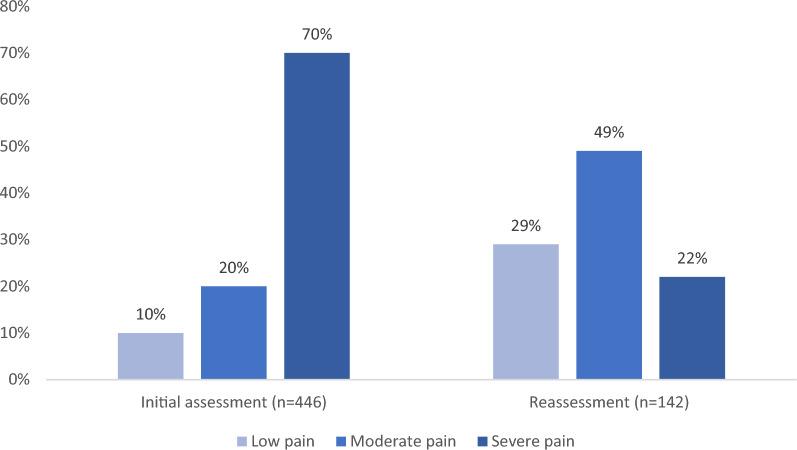
Pain synthesis on initial assessment and reassessment after treatment

Pain was reassessed in 50% (*n* = 158) of the cases where pain treatment was administered. Among those with at least two NRS assessments, 81% (*n* = 92) of the patients experienced a clinically significant pain reduction (≥ 2 NRS units). The pain intensity was reduced by a median of four NRS units (IQR 2.0–5.0) and in pain synthesis (low/moderate/severe) there was a median reduction of one level (IQR 1.0–1.1). See Table 4. Pain synthesis in reassessment and comparison with the initial assessment are illustrated in Fig. 3.

**Table 4:** Reassessment of pain after treatment, pain intensity in reassessment and reduction of pain (n = 317)

Reassessment of pain after treatment	*n*	Percent %	*n* missing
Pain reassessed	158	49.8	0

Pain intensity^a^	Median	IQR	*n* missing
NRS	5.0	3.0–6.0	42

Pain reduction^a^	Median	IQR	*n* missing
Reduction of NRS	4.0	2.0–5.0	44
Reduction of synthesis	1.0	1.0–1.1	16

Clinically significant pain reduction^a^	*n*	Percent %	*n* missing
Cases with ≥ 2 NRS units reduction	92	80.7	44

NRS, Numeric Rating Scale

^a^Among cases with pain reassessment (*n* = 158)

### Low last recording of pain

Among all cases with at least one recorded pain assessment, 19% (*n* = 84) of the patients experienced low pain in the last assessment. Since many cases lacked a recorded pain assessment at all, only 10% of all cases had a last assessment indicating low pain.

## Discussion

Only 10% of the AAP patients left EMS care with pain confirmed to be low. To enable a person-centered approach to pain management, it makes sense not to look at the measures taken by the AC one by one but rather at a chain of actions intended to achieve an acceptable level of pain for the patient during and after the EMS encounter. Our results revealed that only 55% of the patients had a pain assessment recorded, and that only 19% of those patients experienced low pain at the last recording. In total, 90% of the patients left EMS care without pain confirmed to be low meaning they had pain that was either moderate to severe in the last recording, or it was unknown because no assessment had been made and recorded. The cases without an assessment formed a considerable part of the figures at 45%. However, in the context of pain management being discussed as one of the most important contributions of emergency care [9] and pain being a subjective experience, the lack of a recorded pain assessment in itself must be considered highly unsatisfactory in a group of patients suffering from a pain-related condition such as AAP. A previous study revealed that EMS patients who experienced effective pain management rated the overall quality of care better than those who did not [33].

The high proportions of patients with severe (70%) or moderate (20%) pain in the initial assessment and the median initial NRS score (8.0) indicate that the prehospital AAP patients in our study seemed to have considerably more pain than previously described, which further underlines the importance of pain management. In Magnusson et al.’s cohort of AAP patients, only 27% reported severe pain [34], whereas studies including patients in other conditions reported severe pain in 15–40% of the patients [4, 5, 19], with an initial NRS score of 5–5.5 [4, 35]. An explanation for the higher initial pain intensity could be selection bias, if the low proportion of pain assessment is caused by a tendency to assess and document pain in only the most severe cases. The proportions of pain assessment (55%) and NRS scale usage (42%) reported can be compared with those in a previous study, where pain assessment rates as high as 98% in patients with AAP were reported [34]. In other conditions, proportions of assessments ranging from 58% to 95% have been described, and NRS scale usage ranges from 32% to 75% [4, 5, 19, 34–39]. Our method does not allow us to determine whether the low proportion of pain assessments in this study is due to an actual lack of assessments being performed or a tendency to omit documentation of assessments that were in fact conducted into the EPR. Although research on the quality of prehospital documentation remains limited, simulations have shown that there may be substantial room for improvement in the documentation of both pain assessments and other key aspects, such as vital signs [40, 41]. A perceived lack of meaning and purpose in documentation, the prioritization of patient care over record-keeping, and the tendency to document retrospectively rather than in real time have all been identified as potential barriers [41]. Ultimately, the absence of a documented assessment impedes the evaluation of treatment and the detection of improvement or deterioration later in the care pathway, and must be considered suboptimal regardless of the underlying cause.

A commonly discussed way of improving the rate of pain assessment is education [13, 42–44]. However, educational efforts might not have satisfactory effects on assessment, and if results are achieved, they may not be long-lasting [42, 44]. In intensive care unit settings, high workload and lack of priority have been identified as other barriers to pain assessment [45]. Considering the complex process of clinical reasoning in prehospital care [46], similar factors may contribute to pain assessment and documentation being missed in time-pressed situations within the EMS. Previous studies suggest that pain is prevalent in 30–42% of all patients calling for EMS assistance, and there may also be a substantial number of cases where pain is neither assessed nor documented [4, 37]. ACs may use the patient record to guide which questions to ask and which examinations to perform, and they may also rely on mandatory fields to determine whether the record is complete [41]. Consequently, mandatory pain assessment might be one way to increase pain assessment rates, as has also been suggested in previous studies [37, 47]. This, however, requires the provision of adequate scales, as mandatory assessment with inadequate scales may lead to misleading assessments being documented in the lack of adequate ones. Greater agreement between patient and nurse assessments has been reported when a validated scale is used [13]. Scales for self-assessment should be the first choice, as AC pain assessments often result in underestimations. This seems to be especially true for patients suffering from abdominal, traumatic [48] or more severe pain [48–50]. For patients with reduced cognitive ability, the usability of the NRS scale may be reduced [51]. Behavioral scales such as the Behavior Rating Scale (BRS) [52] have been described as feasible alternatives [53]. To facilitate assessment, it makes sense to provide at least one tool for self-assessment and one tool for behavioral assessment in EMS guidelines.

Despite low proportions of pain assessment, we found that patients were receiving prehospital pain treatment to an extent (39%) slightly above previous findings in AAP patients (34%) [34]. From a broader perspective including patients with different types of pain, studies indicate that the proportions of patients receiving prehospital pain treatment range from 8% to 73% [4, 5, 19, 36, 54]. Patients with moderate or severe pain are particularly interesting since their pain exceeds the commonly accepted NRS ≥ 4 threshold for pain treatment, which was also the threshold in the local EMS guidelines. In that group, 62% of the patients received pain treatment. While the fact that two out of five patients with moderate or severe pain were not receiving pain treatment indicate clear room for improvement, it is not surprising considering previous results [19, 53, 54]. A study on patients with suspected acute myocardial infarction reported low adherence to guidelines in terms of pharmacological treatment, including pain treatment with glyceryl trinitrate (53%) and oxycodone (39%) [55]. In part, the low proportions of guideline adherence and pain treatment may be explained by factors such as short prehospital times, contraindications, or missed documentation. Earlier studies have also identified a tendency to sometimes distrust patients’ ratings via pain scales [43, 56], possibly explained by varying and suboptimal ways of explaining the upper limit of the scale [57]. Another barrier could be the concern for masking symptoms, which has been reported in several studies [20, 43, 56]. Despite current evidence showing that early analgesia does not complicate or delay diagnostics [20], the outdated notion that withholding pain management prevents diagnostic errors in cases of abdominal pain may still be prevalent. This belief could contribute to avoidable suffering and complications. It might also partly explain our findings, indicating that educational efforts to debunk this myth could help improve guideline adherence. However, the scarcity of recent studies on pain management barriers within EMS limits our ability to fully assess the relevance of this phenomenon in the context of our findings. Aside from the mentioned factors, low levels of pain treatment may also be related to the subjective nature of pain and a person-centered approach. In such an approach, each patient’s pain and need for treatment is individually evaluated in further depth than just static values. The cutoff points for mild, moderate, and severe pain may vary among individuals [58], and results from postoperative care show that some of the patients do not desire treatment even when the NRS score is four or more [59]. Consequently, full adherence to guideline thresholds would result in a risk of overtreatment, which is likely undesirable. In contrast, a previous study included a specific question to the patients whether they considered their pain “unbearable”. This was the case in 11,8% of the cases, which to a very high extent correlated with the 11,8% of the patients who rated their pain as seven or more on the NRS scale. It cannot be established whether those two groups consisted of exactly the same patients. However, the authors also found the mean NRS score for unbearable pain to be 7.7 ± 1.8, while the mean NRS score for bearable pain was 3.3 ± 2.0 [48]. This could be interpreted that the thresholds for severe pain at NRS ≥ 7 and threshold for treatment at NRS ≥ 4 is reasonable in areas like guidelines and quality assurance. To overcome barriers, widened strategies for pain assessment, more options for pharmacological as well as nonpharmacological treatment and enhanced communication regarding pain management in the chain of care could be considered [43]. However, as with assessments, improvements may be demanding both to achieve and to maintain over time [42].

The most popular medications and administration routes were morphine i.v. and paracetamol i.v., which were used for 20% and 17% of the patients, respectively. Summarizing the use of morphine, alfentanil and sufentanil, the proportion of patients receiving opioid treatment was 23% which is close to previous findings in patients with AAP or digestive symptoms [5, 34]. Opioids have been found to be effective for prehospital pain treatment, with morphine resulting in a ≥ 30% reduction of the initial NRS score in 82% of the patients [60]. To the best of our knowledge, the relatively high usage of paracetamol i.v. in the prehospital treatment of AAP patients has not been reported before but is in line with the EUSEM guidelines for severe pain as an additional treatment besides morphine, ketamine or fentanyl. For mild or moderate pain, paracetamol p.o. is advised by EUSEM [15], but in patients with AAP, factors such as vomiting or the need for surgery may speak for intravenous administration even in some of those cases. The high usage of paracetamol i.v. could be seen as an argument for more EMS organizations to consider it and for further studies to be performed. It might also be a usable supplement for the patients who do not desire opioids, even those with pain exceeding four on the NRS scale [59].

Esketamine usage was very low (1%), which could be related to both pain management guidelines and traditions. Opioids are often the drugs of choice for AAP that is not suspected to be caused by biliary or renal colic. Both esketamine and ketamine have a fast onset and low risk of serious adverse effects, such as respiratory depression or hypotension, which are valuable properties in the prehospital setting. They have also been found to be at least as effective as opioids in patients with pain of other etiologies [61–64], as well as in patients with AAP [65]. To widen the pain treatment options, more EMS organizations might wish to consider esketamine or ketamine as options for AAP patients.

Only half of the patients who received pain treatment were reassessed for pain. While the proportion is higher than previous results ranging from 24–31% [19, 34], it’s still low considering the EUSEM guidelines which advise reassessment once pain treatment has been provided and regularly following that [15]. Mean pain reduction has also been suggested as a quality outcome measure of EMS care, which obviously requires pain reassessment [10].

Among cases where the patient was reassessed for pain, 81% had significant treatment effect (ΔNRS ≥ 2). The median pain reduction was four units on the NRS scale and one unit in the pain synthesis, e.g. from severe to moderate or from moderate to low. This finding is in line with previous results showing that the administration of prehospital pain medication is associated with significant pain relief upon arrival at the ED [66] and is comparable to the findings of previous studies in terms of pain reduction [19, 35, 60]. The median NRS score at reassessment was five. While most patients still had severe (22%) or moderate (49%) pain, the proportion of patients with severe pain was reduced by 48% as the proportion of patients with moderate pain increased by 29%.

The figures indicate that despite the mediocre proportions of assessment, treatment and reassessment, pain management in the cases where pain was actually assessed, treated and reassessed was fairly effective, indicating the potential of prehospital pain management. Unfortunately, the large number of cases without a pain reassessment hampers the reliability of the results. It is possible that a clinically significant change in pain noticed by the AC results in a greater willingness to record a reassessment into the EPR than if pain is unchanged. In the end, the subjective character of pain makes it unavoidable that pain needs to be reassessed and recorded into the EPR following treatment to enable for both short and long term evaluation of pain management, a matter important not only to the individual patient but also for quality assurance and research.

## Limitations

The retrospective nature and dependency of the EPR records are weaknesses in terms of the validity of this study. As previously discussed, the absence of assessment and treatment may, in fact, be the absence of documentation. As much as 30% of the care provided might not be recorded in the patient record [67]. Retrospectivity, however, limits the risk of over- or underestimation that would have been a factor if for example interviews were to be used. This might be particularly true considering that inadequate pain management could be perceived as not fulfilling the responsibilities of an AC. Additionally, a previous study reported that 15% of all records without a pain scale recording had pain mentioned as a symptom in the text, indicating the consideration of all EPR fields to be a strength [37]. With data collected by only one person, the main author, the risk of abstractor bias and lack of possibility to evaluate interrater reliability is another potential source of error [68]. A previous study emphasized how pain interpretation differs among patients, ACs and researchers [69], and it is reasonable to believe that pain interpretation might also differ between individuals within the latter group. Notably, refusal of treatment was not considered. The potential impact of that is difficult to estimate, but earlier studies indicate that 1.2–11% of the patients may refuse pain treatment [34, 36].

In terms of generalizability, the single-center design of the study must be considered a weakness. The results from previous studies on prehospital pain management are somewhat conflicting, and it can be expected that findings are to a certain extent dependent on contextual factors such as available treatments, treatment traditions, guidelines, legislation, EPR design and AC competence requirements. When discussing different competencies and subgroups, it should be noted that a second article exploring such aspects is being prepared for publication.

## Conclusions

Significant room for improvement in the prehospital management of acute abdominal pain was found. The proportions of pain assessment, treatment and reassessment were low, and nine out of ten patients left prehospital care with unknown, moderate or severe pain. This is unsatisfactory, as effective pain management in the prehospital setting is widely acknowledged as important and described by patients as crucial regardless of other factors. However, among the cases where pain assessment, treatment and reassessment were made and recorded, four out of five patients experienced significant pain relief, which can be seen as an indicator of the potential in better prehospital pain management.

## Data Availability

The underlying data of this study were sensitive and retrieved from printed electronic patient records. After registration into the study database, those were destructed in line with the ethics approval. Data from the study database can be made available upon reasonable request, provided that such access complies with the conditions outlined in the ethical approval.

## Abbreviations

AAP: Acute abdominal pain
BRS: Behavior Rating Scale
ED: Emergency Department
EMS: Emergency medical services
EPR: Electronic patient record
EUSEM: European Society for Emergency Medicine
i.v.: Intravenous (administration)
i.n.: Intranasal (administration)
i.m.: Intramuscular (administration)
LOC: Level of consciousness
NRS: Numerical Rating Scale
p.o.: Per oral (administration)
p.r.: Per rectum (administration)
RR: Respiratory rate
SATS: South African Triage Scale
SpO_2_: Peripheral oxygen saturation
Sys BP: Systolic blood pressure
Temp: Body temperature
VAS: Visual Analogue Scale
WMA: World Medical Association
